# Predictors of post-COVID symptoms in Egyptian patients: Drugs used in COVID-19 treatment are incriminated

**DOI:** 10.1371/journal.pone.0266175

**Published:** 2022-03-31

**Authors:** Ahmed Samir Abdelhafiz, Asmaa Ali, Ayman Mohamed Maaly, Mohamed Anwar Mahgoub, Hany Hassan Ziady, Eman Anwar Sultan

**Affiliations:** 1 Department of Clinical Pathology, National Cancer Institute, Cairo University, Cairo, Egypt; 2 Department of Pulmonary Medicine, Abbassia Chest Hospital, MOH, Cairo, Egypt; 3 Department of Anaesthesia and Surgical Intensive Care, Faculty of Medicine, Alexandria University, Alexandria, Egypt; 4 Department of Microbiology, High Institute of Public Health, Alexandria University, Alexandria, Egypt; 5 Department of Community Medicine, Faculty of Medicine, Alexandria University, Alexandria, Egypt; 6 Department of Laboratory Medicine, School of Medicine, Jiangsu University, Zhenjiang, China; Gulu University, UGANDA

## Abstract

**Objectives:**

COVID-19 is a multisystem disease, and some patients suffer from physical or psychological symptoms for weeks or even months after infection, which is described as post-COVID syndrome. The goal of this study is evaluating the prevalence of post-COVID-19 symptoms among Egyptian patients and detecting the factors associated with the presence of these symptoms.

**Methods:**

An on-line cross-sectional survey using Google Forms was used to conduct the present study, which took place between June and August 2021.

**Results:**

Three hundred and ninety-six participants filled in the survey. The mean age of participants was 41.4 years. Most participants had mild to moderate COVID-19 (81.31%). The prevalence of post-COVID-19 symptoms was 87.63%, where the most frequent symptom was fatigue (60.86%). Female sex, the presence of comorbidities, lower degree of education, longer disease duration, as well as severe and critical forms of the disease were significantly associated with the presence of post-COVID symptoms. Using regression analysis, the predictors of post-COVID symptoms were severe and critical forms of the disease and intake of antibiotics and corticosteroids for treatment of COVID-19.

**Conclusions:**

COVID-19 is followed by high prevalence of post-COVID symptoms. To the best of our knowledge, this is the first study to report the relationship between the use of antibiotics and the development of post-COVID symptoms. We recommend further studies to understand this relationship. We also recommend restricting the use of these drugs to indicated cases according to the international guidelines. More studies are needed to gain better understanding of post-COVID symptoms especially in females.

## Introduction

In March 2020, the World Health Organization (WHO) declared COVID-19 as a pandemic. The disease caused by the severe acute respiratory syndrome coronavirus 2 (SARS-CoV-2) has affected millions of people worldwide and is associated with a wide range of symptoms which can be mild, moderate, or severe illness, which could lead to death [1].

Although COVID-19 affects the respiratory system mainly, it is a truly multisystem disease. Extra-respiratory complications are slightly common, and affect renal, cardiac, gastrointestinal, nervous, endocrine, and musculoskeletal systems [2]. In general, it is believed that COVID-19 is a short-term illness, and most health sources indicate that patients with mild disease recover within two weeks from the onset of the symptoms. However, it has been reported that some patients still have symptoms for weeks or even months after infection. The term ‘Post-acute COVID’ refers to the presence of symptoms three weeks after COVID-19 infection, while ‘Chronic COVID’ describes symptoms persisting for more than 12 weeks after infection [3].

Long term symptoms have been reported in the outbreaks of severe acute respiratory syndrome (SARS) and Middle East respiratory syndrome (MERS). About 25% of survivors of SARS and MERS had decreased lung function, respiratory compromise, and decreased exercise capacity for up to 6 months after discharge from hospitals [4]. Moreover, psychiatric symptoms such as post-traumatic stress disorder (PTSD), depression, and anxiety, were also observed in some of these patients one year after discharge [4]. Some healthcare workers suffered from chronic fatigue syndrome up to 20 months after an acute SARS episode, which prevented them from returning to work [5].

Several physical and psychological problems have been reported in patients suffering from post-COVID-19. Physical symptoms include, among others, tiredness or fatigue, brain fog, palpitations, chest pain, dyspnea, cough, muscle pain, and fever [6, 7]. The neuropsychological manifestations of post-COVID-19 included varying degrees of depression, sleep problems and anxiety, as well as PTSD [8]. Herein, we aim at evaluating the prevalence of post-COVID-19 symptoms among Egyptian patients and detecting the factors associated with the development of these symptoms.

## Methods

### Study design and population

An on-line cross-sectional survey was used to conduct the present study, which took place between June and August 2021. The study population was Egyptian subjects of both sexes who had history of COVID-19 disease. The authors shared the link on different social media platforms including WhatsApp and Facebook groups. A Facebook ad that targeted Egyptian people was also used for a couple of weeks during the recruitment phase of the study.

### Study tool

The survey questionnaire was constructed by the authors after reviewing related literature. The questionnaire covered the socio-demographic characteristics, medical history, self-reported information about the previous COVID-19 infection, and about post COVID-19 symptoms. The questionnaire was developed in Arabic, the native language in Egypt.

A preliminary phase was conducted to assess validity and reliability of the questionnaire before its use. Initially, three consultants of chest and/or ICU diseases were asked to assess the degree to which items in the questionnaires are relevant and can correctly evaluate the post-COVID symptoms. After that, minimal corrections were done. The next step was pretesting of the questionnaire. we included 20 participants who were asked to fill the questionnaire twice three weeks apart. Data were used to assess internal consistency reliability using Cronbach’s alpha as well as test-retest reliability using intra-class correlation coefficient. The results showed adequate internal consistency reliability (With Cronbach’s alpha = 0.81) and the intra-class correlation coefficient was 0.96.

### Data collection

An online survey using Google Forms was created, and participants were invited to complete and submit the form. The process of recruiting participants to share in the survey was conducted through convenient sampling.

### Sampling

The sample size was determined using the Epi Info 7 software based on the expected frequency of post COVID-19 symptoms (persistence of at least 1 symptom) of 87.4% [9] at a 95% confidence interval and a 5% margin of error. Based on these parameters, the calculated minimum sample size was 169 participants.

### Ethical considerations

This study was approved by the Ethics Committee of Faculty of Medicine, Alexandria University (IRB No: 00012098). Respondents’ anonymity and confidentiality of their responses were ensured. Informed consents were obtained from participants after explanation of the purpose of the study using the following statement “Completing this questionnaire and submitting it will be considered as your informed consent to participate in this study", as a first step before proceeding to answer the survey questions. Participants had the right to withdraw from the study before submitting the filled questionnaire.

### Statistical analysis

The statistical analysis was performed using Minitab 17.1.0.0 for windows (Minitab Inc., 2013, Pennsylvania, USA). Continuous data were presented as mean and standard deviation, while categorical data were presented as numbers and percentages. The normality of data was examined using Shapiro Wilk test. Independent t test and chi square test were used for univariate analysis of factors associated with post-COVID19 symptoms. The general linear model (GLM) was used to estimate the association between different demographic characteristics of participants and some risk factors with frequency of post-COVID19 symptoms. Multiple logistic regression models were used to find possible predictors for post-COVID symptoms, where the general linear model was used to identify factors associated with increased number of symptoms and severity of the syndrome, and the logistic regression model was used to identify factors which influenced the occurrence of the event itself (presence or absence of post-COVID syndrome). All tests were two-sided, p was considered significant if ≤ 0.05.

## Results

### Participants’ characteristics and disease related factors

As shown in (Table 1), three hundred and ninety-six participants out of 510 completed the online questionnaire, yielding a response rate of 77.64%. The male to female ratio was 1:3. Most participants received high education; in the form of university or post-graduate studies (55.3 and 35.1%, respectively). About one fourth of participants worked in healthcare related sectors, and more than 1/3 suffered from different types of comorbidities, where the predominant comorbidities were hypertension then diabetes mellitus (DM) (21.21% and 9.6%, respectively). Most participants had mild to moderate disease (81.31%). Seventy-four participants suffered from hypoxia (Oxygen saturation less than 92%) and needed Oxygen (O2) supply, but only 40 of them were admitted to the hospital, either in the ward (28/40) or in Intensive Care unit (ICU) (12/40).

**Table 1:** Characteristics of participants in the study.

Factor	Total (n = 396)	
	Mean (N)	SD (%)
Age	41.402	11.151
**Sex**		
Female	311	78.54
Male	85	21.46
**Residence**		
Rural	44	11.11
Urban	352	88.89
**Education**		
Pre-university	38	9.6
University	219	55.3
Post-university	139	35.1
**Occupation**		
Healthcare-worker	97	24.49
Non-healthcare-worker	299	75.51
**Smoking habits**		
Non-smoker	362	91.41
Smoker	34	8.59
**Comorbidity**		
Total (Yes)	150	37.88
Diabetes mellites	38	9.6
Hypertension	84	21.21
Cardiac disease	16	4.04
Thyroid disease	12	3.03
Chest disease	35	8.84
Autoimmune disease	18	4.55
Malignancy	8	2.02
**Disease duration**		
Less than 2 weeks	144	36.36
More than 2 weeks	252	63.64
**Disease characteristics**		
Mild	186	46.97
Moderate	136	34.34
Severe	60	15.15
Critical	14	3.54
Hypoxia	74	18.69
O2 Supply	74	18.69
**Hospital admission**	40	10.1
Ward	28	7.07
ICU	12	3.03
**Treatment protocol**		
No treatment	12	3.03
Professional treatment	318	80.3
Non-professional treatment	66	16.67

Continuous data are represented as mean and standard deviation (SD), and categorical data as number and percentage (%).

**Fig 1:**
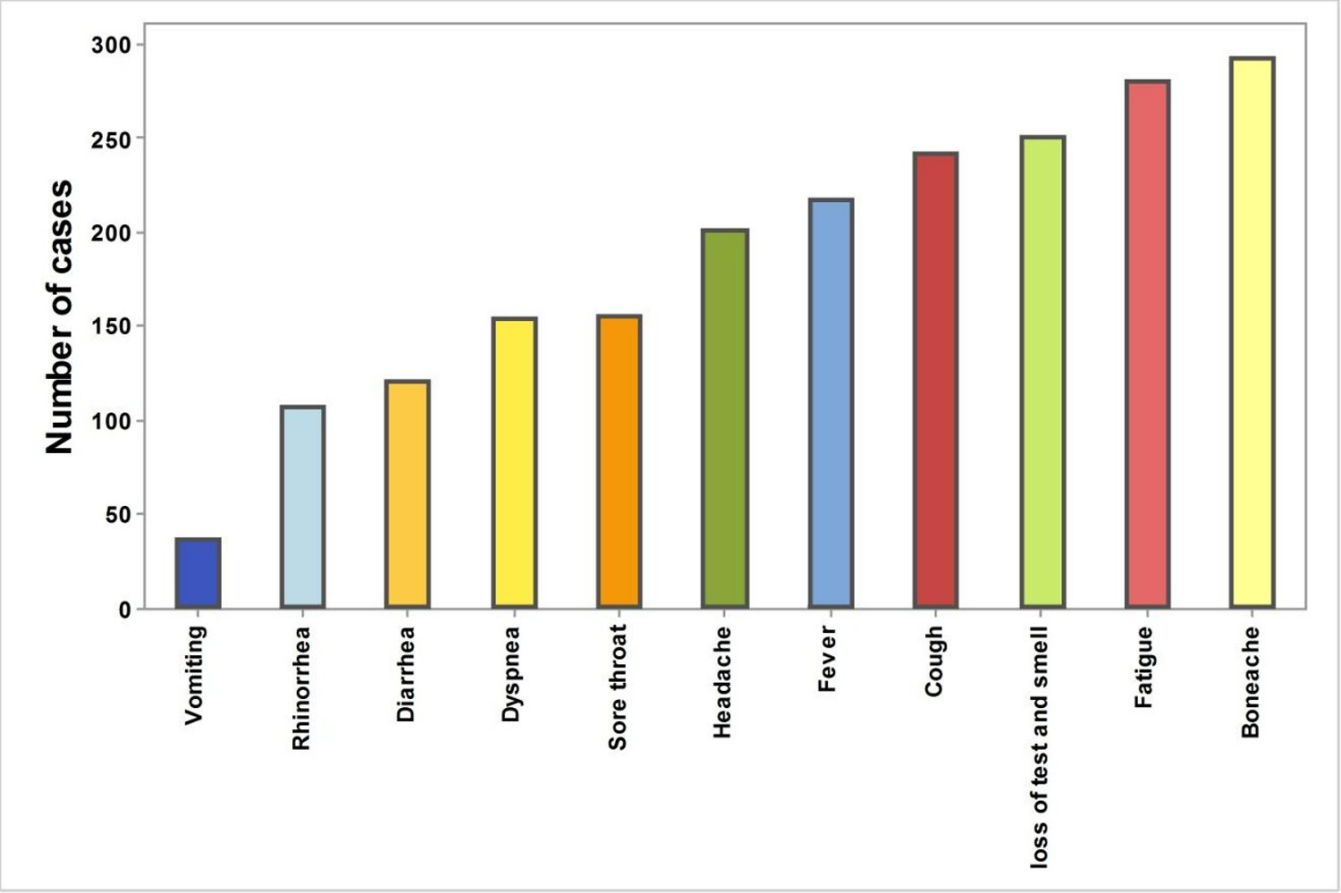
Frequency of different COVID-19 symptoms.

The disease presentation symptoms are presented according to their frequency in (Fig 1 and S1 Table). Common symptoms were bony aches, fatigue and loss of smell and taste (73.99%, 70.96% and 63.38%, respectively).

**Fig 2:**
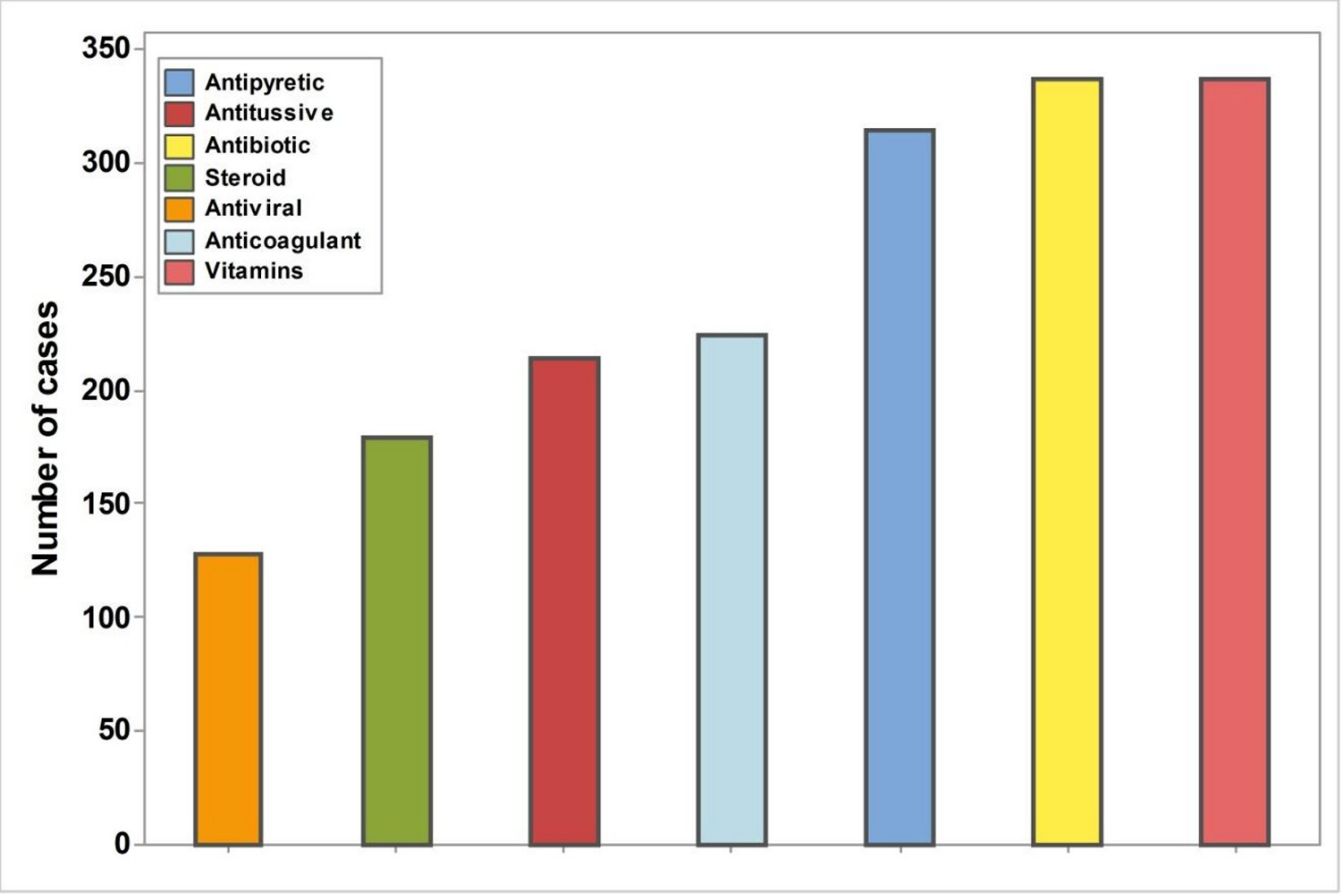
Frequency of drugs used for treatment of COVID-19.

Most participants received professional treatment advised by health care personnel (80.3%), and the most frequently used medications were vitamins, antipyretics, and antibiotics (Fig 2). Corticosteroids and antiviral drugs were used in less than 50% of cases (45.2% and 32.32%, respectively).

## Prevalence of post-COVID-19 symptoms and factors associated with their presence

The prevalence of post-COVID-19 symptoms was 87.63% in our participants, where the most frequent symptom was fatigue (60.86%). The mean (SD) number of post-COVID symptoms was 5, with a range from 0 to 24. Frequency of different symptoms is presented in S2 Table.

As shown in Table 2, female sex, the presence of comorbidities, lower education, longer disease duration, and severe and critical disease were significantly associated with the presence of post-COVID symptoms, (P = 0.04, 0.01, 0.001, < 0.001, and < 0.001 respectively). The frequency of hypoxic cases who needed oxygen supply was greater in the group with post-COVID symptoms, but the difference did not reach a significant value (P = 0.08). Concerning the effect of medications on post-COVID symptoms, results showed that the use of antibiotics, antiviral, anticoagulants, and corticosteroids in treatment was a significant factor for the development of these symptoms (P = 0.001, 0.02, 0.003 and < 0.001 respectively).

**Table 2:** Factors associated with the development of post-COVID symptoms.

Factors	No symptoms (n = 49)		Positive symptoms (n = 347)		P value
	Mean (N)	SD (%)	Mean (N)	SD (%)	
Age	40.5	10.4	41.5	11.3	0.53
**Sex**					
Female	33	67.35	278	80.12	**0.04** ^**#**^
Male	16	32.65	69	19.88	
**Residence**					
Rural	4	8.16	40	11.53	0.48
Urban	45	91.84	307	88.47	
**Education**					
Pre-university	2	4.08	36	10.37	**0.001** ^**#**^
University	18	36.73	201	57.93	
Post-university	29	59.18	110	31.7	
**Occupation**					
Healthcare-worker	21	42.86	76	21.9	**0.001** ^**#**^
Non-healthcare-worker	28	57.14	271	78.1	
**Smoking habits**					
Non-smoker	42	85.71	320	92.22	0.12
Smoker	7	14.29	27	7.78	
**Comorbidity**					
(Yes)	11	22.45	139	40.06	**0.01** ^**#**^
Diabetes mellitus	3	6.12	35	10.09	0.35
Hypertension	6	12.24	78	22.48	0.08
Cardiac disease	0	0	16	4.61	**0.03** ^**#**^
Thyroid disease	0	0	12	3.46	0.07
Chest disease	4	8.16	31	8.93	0.85
Autoimmune disease	2	4.08	16	4.61	0.86
Malignancy	0	0	8	2.31	0.61
**Disease duration**					
Less than 2 weeks	31	63.27	113	32.56	**< 0.001** ^**#**^
More than 2 weeks	18	36.73	234	67.44	
**Disease severity**					
Mild	38	77.55	149	42.94	**< 0.001** ^**#**^
Moderate	7	14.29	137	39.48	
Severe	4	8.16	47	13.54	
Critical	0	0	14	4.03	
Hypoxia	5	10.2	69	19.88	0.08 ^**#**^
O2 Supply	5	10.2	69	19.88	0.08 ^**#**^
Hospital admission	4	8.16	36	10.37	0.61
**Medication**					
Antipyretics	40	86.96	274	80.59	0.27
Antitussives	22	44.9	192	55.33	0.17
Antibiotics	34	69.39	303	87.32	**0.001** ^**#**^
Corticosteroids	8	16.33	171	49.28	**< 0.001** ^**#**^
Antiviral drugs	9	18.37	119	34.29	**0.02** ^**#**^
Anticoagulant	18	36.73	206	59.37	**0.003** ^**#**^
Vitamins	38	77.55	299	86.17	0.11
**Return to regular life**					
Within 2 weeks	31	63.27	107	30.84	**< 0.001** ^**#**^
within 1 month	14	28.57	118	34.01	
More than 1 month	4	8.16	122	35.16	

Continuous data are represented as mean and standard deviation (SD), and categorical data as number and percentage (%)

#: Chi square test, P considered significant if < 0.05.

Severe post-COVID symptoms were significantly associated with younger age, female sex, and longer disease duration (P = 0.001, 0.001, 0.05 respectively) (Table 3). The severity of post-COVID symptoms also increased significantly following the use of antibiotics, corticosteroids, and antiviral drugs (P = 0.02, 0.01 and 0.04, respectively).

**Table 3:** Factors associated with severe post COVID symptoms.

Factors	Coefficient	P value*
Age	**-0.06**	**0.001**
Disease severity	0.70	0.133
Sex (Female)	**0.91**	**0.001**
Residence (Rural)	-0.38	0.257
Occupation (healthcare)	**-0.74**	**0.006**
Smoking habits (Non-smoker)	-0.35	0.353
Comorbidity (No)	-0.34	0.131
Disease duration (< 2 weeks)	**-0.45**	**0.051**
Hypoxia (No)	-0.17	0.679
Hospital admission (No)	-0.20	0.581
Antipyretic (No)	0.25	0.353
Antitussive (No)	-0.17	0.427
Antibiotic (No)	**-0.71**	**0.026**
Corticosteroids (No)	**-0.59**	**0.017**
Antiviral (No)	**-0.49**	**0.041**
Anticoagulant (No)	-0.08	0.734
Vitamins (No)	-0.03	0.902

*: General linear model, the sign before coefficient denotes the direction of correlation, P < 0.05 was considered significant.

### Predictors of post-COVID symptoms

As shown in (Table 4), the predictors of post-COVID symptoms were severe form of the disease, pre-university, and university education, as well as the intake of antibiotics and corticosteroids for treatment, where the development of post-COVID symptoms increased 3 folds with every grade of disease severity and in participants who received pre-university and university education; (OR = 2.85, 3.27 and 2.8, P = 0.01, 0.05 and 0.05, respectively). Additionally, the development of post-COVID symptoms increased up to 2 and 4 folds respectively with administration of antibiotics and corticosteroids (OR = 2.03 and 4.08, P = 0.01 for both).

**Table 4:** Predictors of post COVID symptoms.

Factors	OR	95% CI	P
**Age**	1.00	(0.9618,1.0382)	0.97
**Disease severity**	2.85	(1.2114,6.7222)	0.01
**Sex**			
**Female**	Reference		
**Male**	0.49	(0.2097,1.1554)	0.11
**Residence**			
**Urban**	Reference		
**Rural**	0.62	(0.1501,2.5205)	0.48
**Education**			
**Post-university**	Reference		
**Pre-university**	3.27	(0.5626,19.0377)	0.05
**University**	2.80	(1.1831,6.6155)	0.05
**Occupation**			
**Non-healthcare-worker**	Reference		
**Healthcare-worker**	1.45	(0.6095,3.4459)	0.40
**Smoking habits**			
**Non-smoker**	Reference		
**Smoker**	0.80	(0.2434,2.6245)	0.71
**Comorbidity**			
**No**	Reference		
**Yes**	2.21	(0.9250,5.2595)	0.07
**disease duration**			
**Less than 2 weeks**			
**More than 2 weeks**	2.13	(0.9846,4.5965)	0.05
**Hypoxia**			
**No**	Reference		
**Yes**	0.21	(0.0402,1.0508)	0.06
**Hospital admission**			
**No**	Reference		
**Yes**	0.43	(0.0946,1.9425)	0.28
**Antipyretic**			
**No**	Reference		
**Yes**	0.41	(0.1450,1.1609)	0.08
**Antitussive**			
**No**	Reference		
**Yes**	0.79	(0.3794,1.6464)	0.53
**Antibiotic**			
**No**	Reference		
**Yes**	2.03	(0.8300,4.9758)	0.01
**Steroid**			
**No**	Reference		
**Yes**	4.08	(1.4233,11.7003)	0.01
**Antiviral**			
**No**	Reference		
**Yes**	0.91	(0.3489,2.3629)	0.84
**Anticoagulant**			
**No**	Reference		
**Yes**	0.82	(0.3565,1.8776)	0.64
**Vitamins**			
**No**	Reference		
**Yes**	0.99	(0.3749,2.6253)	0.99

Goodness of fit test: Hosmer-Lemeshow, X^2^ = 3.3, P = 0.9, OR: odds ratio (crude OR), CI: Confidence interval, P values < 0.05were considered as significant values.

## Discussion

COVID-19 is a novel disease; hence little is known about post-COVID symptoms. Post-COVID syndrome represents a group of symptoms that may persist for more than three weeks from the beginning of acute COVID infection. These manifestations could last up to six months or even more after resolution of infection with SARS-CoV-2 [10, 11].

In the current study, 87.63% of participants experienced one or more post-COVID symptoms. This is concordant with other studies where the prevalence of post-COVID syndrome ranged from 87 to 94% [12–14]. Nevertheless, the frequency of post-COVID symptoms was significantly higher among participants who suffered from severe and critical COVID-19 disease and among those with longer disease duration. This coincides with previous studies which reported about 35% prevalence in patients treated for COVID-19 on outpatient basis, but around 87% among hospitalized patients [13, 14].

Subsequently, the probability of post-COVID symptoms was 2 and 4-fold higher with antibiotics and corticosteroids treatment, respectively. This finding agrees to Zha et al. who reported a longer duration of symptoms among patients who received corticosteroids compared to other COVID patients [15]. Moreover, antiviral and anticoagulant therapy were significantly associated with persistence of COVID symptoms. This is expected as these drugs are prescribed for severe and critical cases.

Previous studies reported that corticosteroid therapy may prolong the duration of hospital stay and viral shedding in COVID-19 patients [16]. Yuan et al. found that a significantly higher percentage of COVID patients who received corticosteroids deteriorated to critical conditions compared to the control group [17].

It should be noted that we observed an abuse using corticosteroids and antibiotics for treatment of COVID-19 in Egyptian patients treated at home, even in in the absence of proper indications. Unnecessary use of antibiotics for prophylaxis or treatment of COVID-19 could flare antibiotic resistance. Moreover, antimicrobials such as the macrolide azithromycin, which is widely used for treatment of COVID-19 in Egypt, could also exaggerate the problem. To the best of our knowledge, this is the first study to report such relationship between the use of these drugs and the development of post-COVID symptoms. We think that the side effects of these drugs may contribute to the delayed recovery in those patients. We recommend further studies to understand this relationship. We also recommend restriction of use of these drugs to indicated cases according to international guidelines.

Persistence and severity of post-COVID-19 symptoms were also related to severity of COVID-19 symptoms in our study. The relation between the presence of these symptoms and the severity of acute COVID-19 disease has been proven in several studies [18–21], while other studies failed to identify a significant association between these factors [22–26].

COVID-19 is associated with immune dysregulation, which is related to the severity of the disease [27]. Restoration of baseline and homeostasis after COVID-19 needs some time, which is expected to be longer in severe forms of the diseases. Hospitalization and ICU admission expose patients to long-term sequelae due to extensive tissue damage [28–30]. More specifically, high levels of inflammatory markers and lymphopenia related to the severe condition may explain the persistence of symptoms [30–35]. In general, organ damage, inflammatory response, immune mechanisms, re-infection, complication of comorbidity or adverse effect of medications are thought to be the causes of post-COVID syndrome [36–40]. All these factors are more prevalent in patients with severe disease, which explain the persistent and severe forms of the symptoms in this group.

Although male sex was associated with higher risk of severe COVID-19 [41], we found that female sex was a significant risk factor for post-COVID syndrome. This finding is consistent with Poyraz et al. and Nabavi, who also found that the risk is more common in women compared to men [26, 42]. Women are more likely to develop anxiety, depression, and emotional distress following exposure to significant stress [26]. This psychological effect could be associated with physical symptoms, which should be investigated to gain better understanding of post-COVID symptoms in females.

A wide range of symptoms was reported by our study group, including fatigue, headache, dizziness, myalgia, dyspnea, chest pain, sore throat, persistent loss of taste and smell and others. This range of symptoms is similar to the syndrome previously reported after SARS [43, 44]. Post-COVID fatigue was the most frequently reported symptom by our participants (about 60%). This finding was consistent with studies in several countries [9, 12, 45]. In a review by Pavli et al., the incidence of fatigue reached up to 72% in patients admitted to ICU [46]. Similarly, in China, Huang et al. reported that most patients developed muscle ache, arthralgia, weakness, fatigue, or myalgia following recovery from COVID-19 infection [47]. However, it should be noted that this symptom may be closely related to other symptoms and misinterpretation by the patient is possible. Thus, what the patient exactly feels is the issue. Therefore, we believe that a multidisciplinary approach is needed for follow-up and long-term monitoring of survivors of COVID-19 for evaluation and management of post-COVID symptoms.

## Conclusions and recommendations

COVID-19 is followed by high prevalence of post-COVID symptoms. Long term follow-up for COVID-19 patients is required to understand and deal with the persistent physical and psychological problems in this category of patients. We recommend further studies to understand the relationship between the use of drugs especially antibiotics and corticosteroids and the development of post-COVID symptoms. We also recommend restricting the use of these drugs to indicated cases according to international guidelines. More studies are needed to gain better understanding of post-COVID symptoms in females.

### Limitations of the study

The present study is challenged by some limitations. First, the convenient sampling method used for data collection is not illustrative of all post-COVID patients. To study different patterns of symptoms, it would be more appropriate to include a higher percentage of patients who survived severe or critical COVID-19 illness. Second, the inability to conduct face-to-face interviews limited the chance for clarification of questions for better response rate, better description of symptoms or possible review of records. We tried to explain the meaning of the terms and put examples of commonly used drugs. Even though, we cannot be totally confident of the responses. Lastly, follow-up studies of patients for longer periods would help further assessment of persistent symptoms.

## Supporting information

S1 Table Frequency of COVID-19 symptoms. *Participants presented with one or more than one symptom.
(DOCX)

S2 Table Frequency of different post-COVID symptoms. Continuous data represented as mean and standard deviation (SD), and categorical data as number and percentage (%). *Participants presented with one or more than one symptom.
(DOCX)
