# Correction: Predictors of post-COVID symptoms in Egyptian patients: Drugs used in COVID-19 treatment are incriminated

**DOI:** 10.1371/journal.pone.0271154

**Published:** 2022-07-05

**Authors:** The PLOS ONE Staff

There are errors in the author affiliations. The publisher apologizes for the errors. The correct affiliations are as follows:

Ahmed Samir Abdelhafiz^1^, Asmaa Ali^2,6^, Ayman Mohamed Maaly^3^, Mohamed Anwar Mahgoub^4^, Hany Hassan Ziady^5^, Eman Anwar Sultan^5^

**1** Department of Clinical Pathology, National Cancer Institute, Cairo University, Cairo, Egypt, **2** Department of Pulmonary Medicine, Abbassia Chest Hospital, MOH, Cairo, Egypt, **3** Department of Anaesthesia and Surgical Intensive Care, Faculty of Medicine, Alexandria University, Alexandria, Egypt, **4** Department of Microbiology, High Institute of Public Health, Alexandria University, Alexandria, Egypt, **5** Department of Community Medicine, Faculty of Medicine, Alexandria University, Alexandria, Egypt, **6** Department of Laboratory Medicine, School of Medicine, Jiangsu University, Zhenjiang, China
