# Supplementary material for: Predictors of post-COVID symptoms in Egyptian patients: Drugs used in COVID-19 treatment are incriminated
Source: PLoS One. 2022 Mar 31;17(3):e0266175. doi: 10.1371/journal.pone.0266175 (PMC8970499; doi:10.1371/journal.pone.0266175)
Supplement: S1 Table — *Participants presented with one or more than one symptom. (DOCX) [file pone.0266175.s001.docx]

Supplementary files

**Supplementary table (1): Frequency of COVID-19 symptoms**

| **Symptom*** | **Total (n=396)** | |
| --- | --- | --- |
|  | **N** | **%** |
| Fever | 218 | 55.05 |
| Cough | 242 | 61.11 |
| Dyspnea | 154 | 38.89 |
| Headache | 201 | 50.76 |
| Sore throat | 155 | 39.14 |
| Rhinorrhea | 107 | 27.02 |
| Fatigue | 281 | 70.96 |
| Bone ache | 293 | 73.99 |
| loss of test and smell | 251 | 63.38 |
| Diarrhea | 121 | 30.56 |
| Vomiting | 36 | 9.09 |

*Participants presented with one or more than one symptom
