# Supplementary material for: Predictors of post-COVID symptoms in Egyptian patients: Drugs used in COVID-19 treatment are incriminated
Source: PLoS One. 2022 Mar 31;17(3):e0266175. doi: 10.1371/journal.pone.0266175 (PMC8970499; doi:10.1371/journal.pone.0266175)
Supplement: S2 Table — Continuous data represented as mean and standard deviation (SD), and categorical data as number and percentage (%). *Participants presented with one or more than one symptom. (DOCX) [file pone.0266175.s002.docx]

Supplementary files

**Supplementary table (2): Frequency of different post-COVID symptoms**

|  | Total (n=396) | |
| --- | --- | --- |
| **Symptoms*** | **N** | **%** |
| Yes | 347 | 87.63 |
| Fatigue | 241 | 60.86 |
| Loss of concentration | 144 | 36.36 |
| Confusion | 97 | 24.49 |
| Headache | 91 | 22.98 |
| Long lasting loss of taste/smell | 67 | 16.92 |
| Parosmia | 51 | 12.88 |
| Vertigo | 63 | 15.91 |
| Tinnitus | 62 | 15.66 |
| Palpitations | 112 | 28.28 |
| Sore throat | 35 | 8.84 |
| Muscle/joint pain | 158 | 39.9 |
| Chest pain | 108 | 27.27 |
| Dyspnea | 84 | 21.21 |
| Nausea | 25 | 6.31 |
| Cough | 46 | 11.62 |
| Sweating | 58 | 14.65 |
| Fever | 11 | 2.78 |
| Abdominal pain | 46 | 11.62 |
| Neuralgia | 84 | 21.21 |
| Skin allergy | 27 | 6.82 |
| Diarrhea | 28 | 7.07 |
| Thrombotic events anywhere in the body | 3 | 0.76 |
| Depression stress and anxiety | 115 | 29.04 |
| Urinary tract infection | 95 | 23.99 |
| Insomnia | 20 | 5.05 |
| Chills | 87 | 21.97 |
| No symptoms | 39 | 9.85 |
| Number of symptoms | Mean | SD |
|  | 5.247 | 4.381 |
|  | Min | Max |
|  | 0 | 24 |

**Continuous data represented as mean and standard deviation (SD), and categorical data as number and percentage (%)**

***Participants presented with one or more than one symptom**
